# Effect of Ziziphus and Cordia Gums on Dough Properties and Baking Performance of Cookies

**DOI:** 10.3390/molecules27103066

**Published:** 2022-05-10

**Authors:** Abdellatif A. Mohamed, Mohamed Saleh Alamri, Shahzad Hussain, Mohamed A. Ibraheem, Akram A. Abdo Qasem, Ghalia Shamlan, Ibrahim A. Ababtain

**Affiliations:** Department of Food Science and Nutrition, King Saud University, Riyadh 1145, Saudi Arabia; msalamri@ksu.edu.sa (M.S.A.); shhussain@ksu.edu.sa (S.H.); mfadol@ksu.edu.sa (M.A.I.); aqasem@ksu.edu.sa (A.A.A.Q.); shamlana@ksu.edu.sa (G.S.); ababtain.ibr@gmail.com (I.A.A.)

**Keywords:** cookies, texture, gum, cordia, ziziphus, sensory

## Abstract

The influence of 2% and 5% Cordia (CG) and Ziziphus (ZG) gums on dough characteristics and cookie quality was investigated. Micro-DoughLab, a texture analyzer (TA), a rapid viscoanalyzer (RVA), and solvent retention capacity were used to examine the effect of CG and ZG gums on dough physicochemical parameters (SRC) and cookie quality. The diameter, thickness, spread, and sensory evaluation of cookies were evaluated. With the addition of CG and ZG, dough softness, mixing time, and mixing tolerance index (MTI) increased, whereas stability and water absorption decreased. TA data showed that adding gums resulted in softer and less sticky doughs than the control, whereas RVA data showed that adding CG resulted in a significant increase in peak viscosity, but no change in flour gel setback. In comparison to the control and CG samples, the ZG samples exhibited the most dough extensibility. The thickness and diameter of the cookies increased but the spread decreased, due to the added gums. The gum-containing cookies had a lower overall acceptability by panelists than the control, although only by a small margin. Gum-containing cookies, on the other hand, can deliver up to 5% soluble fiber.

## 1. Introduction

Hydrocolloids, primarily water-soluble polysaccharides from various sources, can be added to wheat flours to improve breadmaking quality, and to optimize gluten-free bread formulations [1,2]. Because these molecules do not or only partially degrade in the digestive tract, they may contribute significantly to the overall fiber content of flours, depending on the level used. Although bacterial gums, such as xanthan, dextran, and gellan, are also utilized in foods, the majority of hydrocolloids used in foods come from plants. In contrast to other additives that have a minor impact on flour water absorption, the addition of these compounds is expected to have a significant impact on this parameter, due to their very hydrophilic properties. Hydrocolloid incorporation caused rheological changes in dough; the amount of water added, as well as the structure and concentration of the hydrocolloid, influenced the trend and magnitude of this effect. When hydrocolloids were added to the dough, it resulted in a softer texture and less cohesive dough than when the control flour was used, both in conditions of water availability and water restriction [3].

The most important ingredient in cookie production is flour, but for commercial cookies, soft wheat flour with a medium gluten protein strength is usually preferred. Sugar and fat are the next most essential elements after flour, with sugar and fat levels in cookies typically being high. Sucrose is the most commonly used sugar in cookie baking, but sugar functionality varies based on sugar type and particle size, both of which are important factors in cookie finished-product quality. In a normal cookie formula, the high sugar concentration formula inhibits gluten development during cookie dough mixing and sheeting, whereas other baked product formulas with lower sugar concentrations allow gluten development to occur during mixing [4]. Despite the widespread use of empirical rheological and baking tests, they all only measure the combined contributions of the major flour functional components, which include damaged starch, gluten proteins, and pentosans, rather than the individual functional contributions of each of those components. Through a greater understanding of dough mixing and cookie/cracker-baking mechanisms, end-users will be able to better predict flour functionality and improve biscuit quality by analyzing the unique functional contributions of each functional component of flour. The solvent retention capacity (SRC) method was designed and developed by [4] as a valuable tool for measuring flour functionality for soft wheat applications. The SRC test is a solvation assay for flours that uses the enhanced swelling behavior of individual polymer networks of soft wheat flour in a single diagnostic solvents-water, 5% *w*/*w* lactic acid in water (for gluten), 5% *w*/*w* sodium carbonate in water (for damaged starch), and 50% *w*/*w* sucrose in water (for pentosans) to predict the functional contribution of each individual flour component. Wheat breeders, millers, and bakers are increasingly using the SRC method, and the association between flour SRC profiles and cookie and cracker quality has recently been widely reported [5,6,7,8,9,10]. The amylose level increased the soft wheat baking quality associated with sugar snap cookie (SSC) diameter. The water SRC test had the highest correlation with amylose content and SSC diameter of the four types of SRC tests (water, sodium carbonate, sucrose, and lactic acid). Only water SRC was substantially associated to amylose content among the four SRC tests when a regression analysis was undertaken, comparing non-waxy and partial waxy isogenic lines accessible in commercial markets. This shows that a high amylose content is essential for improving the quality of soft wheat baking, a procedure that requires reduced water retention capacity [9]. The amount of water in a dough affects its rheology, since a lack of water causes doughs to be overly stiff and difficult to manage, as well as unable to produce a suitable fermentation volume. The inclusion of gums could affect the rheological features of dough in two ways, which are as follows: (1) by requiring more water to achieve the desired consistency of dough, and (2) by the effect of possible interactions between different macro-polymers (proteins of gluten network). Because polysaccharides have varied structures and hydrophilicities, changes in dough rheological behavior should be predicted depending on the kind and concentration of the gum [11].

The major quality criteria for cookie quality are a larger diameter and a higher spread factor [12]. Because of the leavening gases, the dough swells and flows during the baking process. The final cookie size is determined by the rate of flow and the moment when the expansion ceases. Doughs made with high-quality flour flow much faster than doughs made with low-quality flour [13]. The amount of water in the dough and the strength of the dough dictate the flow set time. As a result, the ultimate cookie quality is determined by the chemical constituents in flour that hold water and the quality and quantity of gluten proteins that determine dough strength and extensibility. Cookies are typically made with soft wheats that have low protein content and weak gluten [14]. *Cordia myxa* is a flowering plant that belongs to the *Boraginaceae* family of plants. Cordia gum is an anionic polysaccharide used as an emulsifier and tablet binder with high adhesive qualities. It is also used as an antistaling agent in bread and as a coating for pine nut fruits to keep them from oxidizing. Cordia gum is reported to have a 1.8 million Da molecular weight and the main components of the polymer include galactose (27%), rhamnose (21%), mannose (17%), xylose (11%), glucose (10%), arabinose (9.5%), and uronic acids (5%) [15]. The Rhamnaceae family of plants includes *Zizyphus Spina-Christi*, a tree species. It can be found growing throughout a large area of Africa, from Mauritania to the Red Sea. According to the literature, ethanol extract from Ziziphus fruits has rheological qualities comparable to xanthan gum and superior to guar gum. Ziziphus mucilage should be extracted with 1:7 water at 60 °C and precipitated with 1:3 ethanol. Water holding capacity, oil absorption, and emulsifying ability, respectively, were 73.35 g water/g dry base, 4.97 g oil/g dry sample, and 52.22% of the dry sample [16]. The objectives of this study were to determine the impact of Cordia and Ziziphus gums on the physical changes that occur during dough mixing, as well as their impact on the rheological properties of doughs and the quality of baked cookies. The study also sought to determine the appropriate gum for cookie making.

## 2. Results and Discussion

### 2.1. Pasting Properties

**Figure 1:**
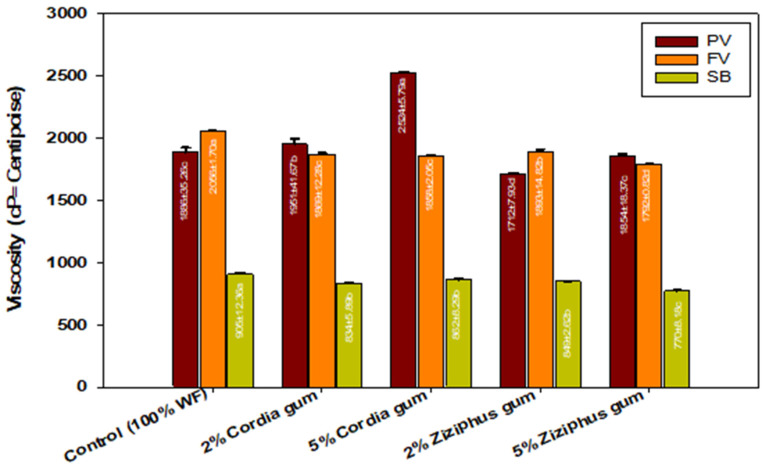
Rapid Visco Analyzer (RVA) pasting properties of wheat flour gels containing different levels of Cordia and Ziziphus gums. PV = peak viscosity; FV = final viscosity; SB = setback viscosity; values followed by different letters in same color bars are significantly different at *p* ˂ 0.05.

Figure 1 shows the pasting properties of the flour and flour mixture gels. Cordia gum (CG) caused a significant rise in peak viscosity (PV) (*p* < 0.05) at 2 or 5% levels, although Ziziphus gum (ZG) had no influence on P.V at either level compared to the control. The extended starch granules’ swelling and the gum’s ability to enhance the concentration of solids in the liquid phase of the slurry during the earliest stages of starch gelatinization could explain the increase in peak viscosity due to CG. In the case of ZG, the gum appears to have inhibited granule swelling by covering the surface of the starch granules, resulting in a lower PV. We previously reported that when CG and ZG were combined with pure starch, the PV increased [17]. When compared to the PV in wheat flour, which was reported here, the PV of pure starch reported by Mohamed et al., (2022) dropped from 2970 cP to 1886 cP as shown in Figure 1, which could be due to gluten interference or dilution of the starch with the gluten. As a result, the presence of wheat gluten in the flour altered the effect of both gums on the PV of starch in the flour, particularly ZG. Because the PV of starch in the flour-ZG blend dropped from 1886 to 1712, it increased from 1886 to 2524 cP for CG, as shown in Figure 1. Both gums greatly reduced the control’s setback (SB) in the following order: control > 2% or 5% CG > 2% ZG > 5% ZG. Amylose retrogradation is the primary cause of SB (amylose hydrogen bonding network). Lower SB suggests less amylose retrogradation due to the week network, which may be attributed to gum–amylose interaction. The gums caused a decrease in final viscosity (FV), with the FV of control > 2% ZG > 5% CG > 2% CG > 5%. Because amylose retrogradation is a cause of a number of quality concerns in baked goods, lower retrogradation is a desirable attribute of the CG and ZC gums.

### 2.2. Wheat Flour Gel Texture

**Figure 2:**
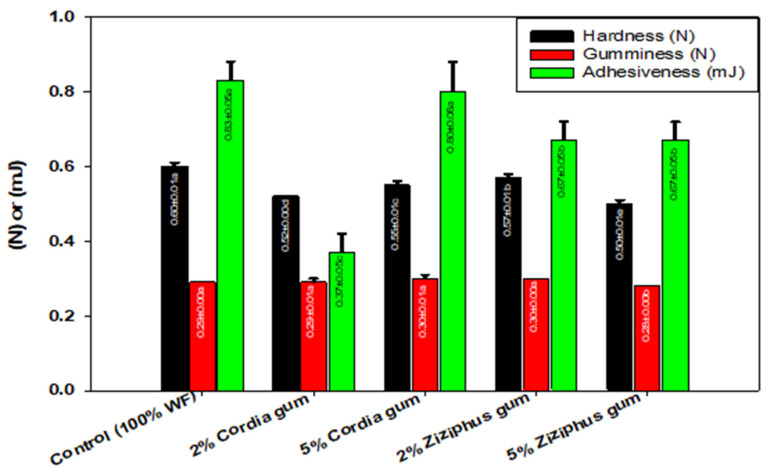
Textural properties of wheat flour dough mixed with Cordia and Ziziphus gum blends. Values followed by different letters in same color bars are significantly different at *p* ˂ 0.05.

The textural parameters, as described in Figure 2, include the hardness, which is the force required for sample deformation; cohesiveness, which is the strength of the internal bonding in the sample; adhesiveness, which is the stickiness of the surface and gumminess, which is the energy required to breakdown semi-solid food in the mouth until it is ready to swallow (hardness × cohesiveness). The texture of the dough was evaluated after it had been stored for 40 min. The dough hardness dropped significantly after both gums were added; however, the decline in hardness depended on the gum type and concentration. Since ZG had a greater influence than CG, the 5% ZG had the greatest decline compared to the 2%. The 2% ZG was less effective than the 5%, whereas the 2% CG was more efficient in reducing hardness than the 5%. It is suggested that the dough’s hardness is due to the formation of a large number of disulfide bonds, although sulfhydryl-disulfide inter-change occurs during the resting period, causing the dough to relax [18]. As a result, when 5% ZG was added, the most disulfide bonds were lost, and when 2% ZG was added, the least bonds were broken. It appears that the gum reduces the mobility of the dough during mixing because it competes with the gluten for the water, which reduces the chances of disulfide bonds formation during mixing and it increases the loss of disulfide bonds during dough resting. As a consequence, free radicals engaged in raising sulfhydryl groups and lowering disulfide bonds appeared to be promoted by the presence of 5% ZG. Except when 5% ZG was added, the gumminess remained unchanged, indicating that the overall effect of the energy required to break down the internal forces of the dough did not vary considerably. Except for the 5% CG, the addition of both gums significantly reduced the adhesiveness, which indicates the dough surface stickiness; nonetheless, CG at 2% had the largest effect. This suggests that, unlike CG, ZG had no concentration-dependent impact. As a result, adding both gums to the dough will have no influence on its stickiness or overall machinability and handling.

### 2.3. Dough Mixing Properties

The dough mixing properties were determined using DoughLab and the results are shown in Table 1. The effects of CG and ZG on the characteristics of wheat flour dough were identified in this investigation. The effect was influenced by the gum type, as well as the concentration. Except for the 2% ZG, there was a significant reduction (*p* < 0.05) in water absorption (WA) due to the addition of the gums, but the effect of the 5% level of both gums was identical, which was lower than the control. The WA changes depending on the gum’s structure and ability to absorb water and interfere with the flour’s ability to absorb water. The reduction in WA caused by CG was identical to that observed in the literature with guar gum [3]. As a result, CG is preferable to ZG, since it reduces the WA and the dough development time compared to the control or the ZG. The amount of time it takes for flour dough to reach its maximum consistency after it has been mixed with water is referred to as dough development time (DDT). CG showed a significant reduction in dough development time (DDT), whereas ZG showed a significant increase in DDT compared with the control (Table 1). Because the reduction in WA caused by both gums (except for 2% ZG) was followed by a significant drop in DDT owing to CG, adding ZG resulted in a concentration-dependent increase in DDT. Although it agrees with the effect of k-carrageenan or methoxy hydrophilic cellulose derivative (HPMC), the effect of CG on DDT shown here contradicts the reports that show that adding hydrocolloids, such as xanthan, alginate, or guar, increases DDT [19]. Evidently, when hydrocolloids are added, it takes longer for the dough matrix to develop, due to their hygroscopicity, resulting in a higher DDT, and the opposite is true when the DDT is reduced. As a result, we can infer that CG is less hygroscopic than ZG and has a plasticizing influence on the formation of gluten networks. Dough stability is a measure for a dough’s ability to maintain consistency over time and a mechanical strength indicator. Both gums significantly reduced dough stability at both concentrations (Table 1); however, the 2% ZG had the smallest decline, 3.50 min vs. 5.70 min for the control. Other gums, such as alginate and xanthan gum, have been shown to decrease the stability of wheat flour dough, but guar gum has been shown to boost dough stability [20]. The mixing tolerance index (MTI) reflects dough softening during mixing as the difference between the BU at the top of the curve at peak time and the value at the top of the curve 5 min later. A MTI value of 30 BU or less is considered exceptionally excellent for bread wheat flours. A flour with an MTI of more than 50 FU has a lower mixing tolerance and is more likely to cause issues during mechanical handling and dough preparation. The addition of both gums reduced MTI, and increasing the gum concentration increased MTI even more, indicating dough softening and decreased mixing tolerance. The detrimental impact of the 2% of both gums on MTI was much smaller than that of the 5% and was concentration dependent, with the 5% CG having a 3-fold increase in MTI compared to the control. Other gums, such as xanthan, guar, and alginate, have been shown to reduce the MTI of strong gluten hard red spring wheat flour at 2% concentrations, with alginate reducing the MTI to 0 at 2% [21]. The FQN is the length from the start of water addition to the point where the center of the curve is 30 units lower than that at the development time. The FQN significantly decreased after both gums were added, although the 5% level of both gums showed the largest reduction (Table 1). This could be related to gum–gluten interaction, as rice bran and bagasse fiber have been shown to reduce wheat flour FQN, due to gluten–fiber interaction. The difference between the consistency value of the dough mixing curve center at the end of the developing time and the curve center 12 min later is the degree of dough softening. Both gums greatly increased dough softening, with the 5% level showing the greatest increase. The following order was used for softening: 5% CG > 5% ZG > 2% ZG > 2% CG > control.

### 2.4. Wheat Flour Dough Extensibility

**Figure 3:**
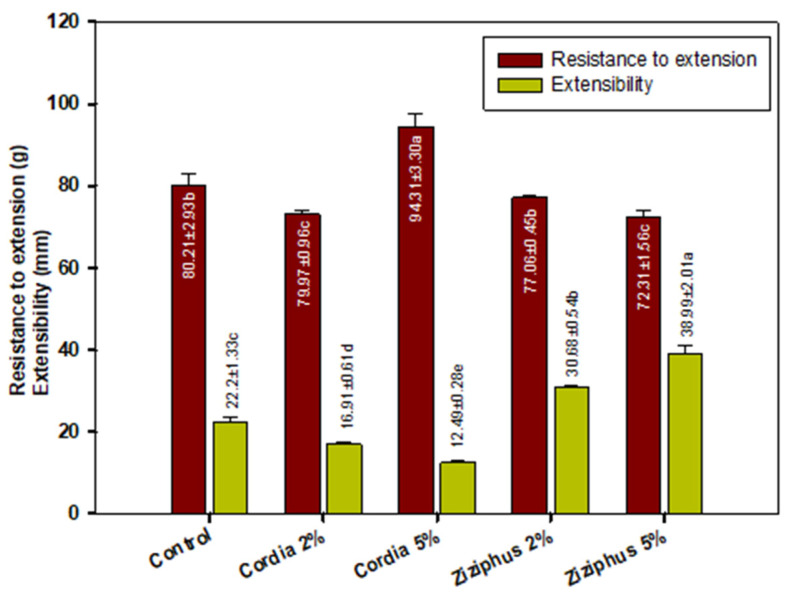
Dough extensibility properties of flour gum blends. Values followed by different letters in same color bars are significantly different at *p* ˂ 0.05.

Figure 3 shows the dough’s resistance to extension and extensibility. The 5% CG was the most resistant to extension, being more resistant than the control, while the control and Ziziphus gum had a similar impact. ZG, on the other hand, significantly increased dough extensibility compared to the control or CG. In the presence of both gums, dough resistance to extension was reflected by the dough hardness, as shown in Figure 2, whereas extensibility was represented primarily by the gumminess of the gels prepared with ZG. Dough extensibility is a measure of the dough’s strength and a factor in the consistency and quality of the finished baked item. Extensibility and elasticity are a complex dough balancing act that starts with mixing and proceeds with gluten matrix development, resulting in extensible and elastic properties, which means it can stretch and return to its original shape. Desirable dough qualities are the consequence of a mix of good resistance and extensibility [20]. The dough matrix is made up of the following two phases: a continuous phase (high molecular weight glutenin), contributed by disulfide bond formation and represented by the elastic property, and a discontinuous phase (low molecular weight gliadin), represented by the viscous property. Gums are positioned in the discontinuous phase, which contributes to the balance between dough extensibility and elasticity, since they are low molecular weight molecules compared to glutenin. The extensibility of cookie dough improved with increasing levels of ZG (Figure 3), but CG provided the least extensibility. The dough with the highest resistance to extension was prepared with 5% CG, which also had the lowest extensibility, indicating a less elastic dough. According to the literature, xanthan gum-containing flour mixes have a reduced dough extensibility [22]. This was in agreement with previous reports that millet flour reduced cookie dough extensibility [23]. As a result, based on the extensibility performance, ZG is better suited for manufacturing cookie dough than CG.

### 2.5. Solvent Retention Capacity (SRC)

Solvent retention capacity of the control flour and the blends is given in Table 2. Solvent retention capacity creates a useful flour quality and functionality profile that assists in predicting the baking performance of flour components for high-quality final products. In general, all water-absorbing components in flour influence water solvent retention capacity (WRC), lactic acid solvent retention capacity (LaSRC) is linked to gluten protein characteristics, sodium carbonate solvent retention capacity (ScSRC) is associated to damaged starch levels, and sucrose solvent retention capacity (SuSRC) is related to pentosans [24]. The WRC increased significantly (*p* < 0.05) with the addition of CG and ZG, and the rise was concentration dependent (Table 2), with the largest WRC recorded for the 5% CG flowed by the 5% ZG, but with no significant difference between the two gums at the 2% level. Similarly, the SuSRC significantly increased by both gums, especially at the 5% level and with the following order: 5% CG > 5% ZG >2% CG > 2% ZG > control. The SuSRC is unique in the sense that it mimics the functional environment in cookie or high sugar cracker dough and gives an indication for the flour pentosans’ characteristics [25]. The blends exhibited the SCRC values as follows: 5% ZG > 5% CG > 2% CG > 2% ZG > control, whereas the LARC values were as follows: 5% ZG > control > 2% CG > 2% ZG > 5% CG. The behavior patterns of the SCRC values are connected to flour quality for baking performance in various end-use applications. Different patterns are best suited to certain products. For example, a cookie flour with a WRC of 51%, sucrose of 89%, lactic acid of 87%, and sodium carbonate of 64% may perform well, whereas a WRC of 57%, SuSRC of 96%, LARC of 100%, and SCRC of 72% may work effectively in a sponge and dough system [25]. According to Guttieri, et al. [26], soft wheats with high LARC values have strong gluten and are suited for crackers and flat bread, while those with low LARC values have weaker gluten and are best suited for pastries [27]. The flour used in this work exhibited 74% WRC, 130% SuSRC, 101% SCRC and 143% LARC. Because of the high LARC, the flour used here fit the profile of a soft wheat suited for flat bread and crackers, but the high SuSRC, which mimics the functional environment in cookies or high sugar crackers, shows that this flour is suitable for cookie production. However, if the SRC values are pattern range rather than a fixed number, flour conformance to bakery manufacturing will improve.

### 2.6. Physical Analysis of Cookies

Table 3 shows the physical examination of the four cookies. The results showed a significant difference (*p* < 0.05) between the control and the blends, with the thickness of the cookies increasing in the sequence of 5% CG > 5% ZG > 2% CG > 2% ZG > control. The dough softening and sugar solvent retention (SuSRC) values, as described in Section 3.4, followed a similar pattern. In Table 1, the blend with 2% ZG exhibited low water holding capacity, which is reflected in the low cookie thickness, as shown in Table 3.

These data indicate that a low water holding value leads to low cookie thickness. This suggests that thicker cookies can be produced with softer dough, which indicates a weakening of the limited gluten network created during dough mixing. A similar pattern was observed for cookie diameter, where the diameter grew larger than the control after the addition of the gum. Cookie diameter and protein content were shown to have an inverse relationship [28]. The creation of a continuous gluten network raises the viscosity of the dough and stop the flow of the cookie [29]. The control had the lowest thickness of 8.0 ± 0.3 mm and the diameter of 53.50 ± 0.50, whereas cookies with 5% CG had the highest thickness of 10.26 ± 0.10 mm and the largest diameter of 56.44 ± 0.21. The addition of both gums significantly decreased the spread ratio of cookies compared to the control. The effect of the amount or the type of gum on the spread showed no significant difference, except for the 2% ZG (Table 3). In general, flours with a low water-retention capacity are considered to be superior for cookie baking [4] because the amount of water in the cookie mix determines the dough’s viscosity. More sugar is dissolved while mixing when there is more water available in the dough. This reduces the initial viscosity of the dough, allowing the cookie to spread more rapidly during baking. The flour components that absorb a lot of water lower the amount of water required to dissolve the sugar. As a result, the initial viscosity of the dough is higher, and the cookie spreads less during baking [29]. As a result, the low cookie spread induced by gum addition was attributable to the gum’s high-water absorption, which resulted in a high dough viscosity, limiting spread.

Cookies hardness is among the parameters that influence the consumer’s acceptability. The research presented here demonstrated a decrease in cookie hardness as a result of the gums, particularly at the 5% addition (Table 4). Increased cookie hardness has been linked in the literature to a smaller spread ratio of cookies and, as a result, a more compact structure, as has been confirmed in previous research [30]. In general, the studies that support the concept that high-absorbing ingredients, such as bran or germinated flour, can induce an increase in cookie hardness contradict the facts presented here. This could be explained by the gum’s capacity to hold water and maintain moisture during baking, resulting in softer cookies. Gum is supposed to increase hardness because it reduces cookie spread, according to the suggestions by previous works [31].

Fracturability increased from 3.93 ± 0.04 to 5.90 ± 0.04 (mm) in the control and 2% ZG, respectively, when gum was added (Table 4). The decrease in hardness and rise in fracturability of the cookies shown here differs from previous reports [32], which is due to the differences in the ingredients added to the base flour.

**Figure 4:**
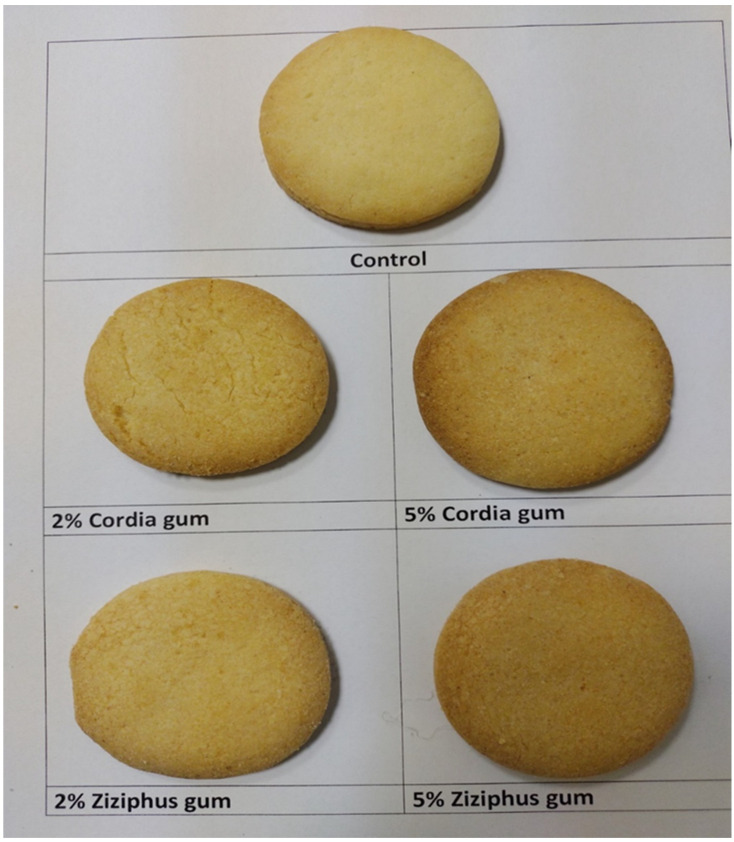
Pictures of cookies prepared from the control (wheat flour) and different blends.

The color of the cookies lightened, lower L*, as the gum content increased, with a higher CG content increasing the brightness of the cookie and a higher ZG content darkening the cookie, but still keeping it lighter than the control (Table 4 and Figure 4). According to Sharma and Gujral [33], baking dough into cookies resulted in a significant shift in color, with lower L* values and higher a* and b* values than the corresponding dough. The 2% CG exhibited the lightest sample after the control, while 2% ZG was the darkest (Table 4).

### 2.7. Sensory Evaluation of Cookies

Table 5 shows the sensory evaluation of the cookies made with wheat flour and the mixtures. The added gums had a significant impact on the taste and aroma, but the score of the texture was greatly enhanced with the addition of Ziziphus gum. The amount of ZG in the cookies raised the sensory scores for texture. The greatest texture score (8.41) was achieved by blending 2% or 5% ZG into the dough, which could be attributed to the plasticizing impact of ZG on the dough that offers uniformity to the cookie texture. This was noticed in the above-mentioned cookie hardness and fracturability tests. The color was also altered, as evidenced by the L* value drop. When compared to the control, the blends had a lower overall acceptance. The color sensory scores improved as the amount of ZG in the cookies increased. The greatest color score (8.30) was achieved by blending 5% ZG, which could be related to the particle size of the ZG that lends uniformity to the cookie color.

## 3. Materials and Methods

### 3.1. Materials

Flour was purchased at a nearby supermarket (Riyadh, Saudi Arabia). Sigma Aldrich provided sodium bicarbonate, lactic acid, and sucrose (St. Louis, MO, USA). The control flour was replaced with 2% or 5% Cordia or Ziziphus gums to make the blends.

### 3.2. Pasting Properties

Rapid Visco Analyzer measurements (RVA, Newport Scientific, Sydney, Australia) were carried out to measure the pasting properties. The flour/gum mixes or the control (3 g at 14% moisture basis) were weighed straight into special RVA canisters, and distilled water was added to make a total weight of 28 g. Wheat flour alone, with no gum, was used as the control. The slurry was heated to 50 °C for 30 s, then to 95 °C in 4.40 min (at 10.23 °C/min) and held for 4 min. The sample was cooled to 50 °C in 4 min and then kept at 50 °C for 2 min [33]. The peak viscosity of the produced gel, final viscosity, and setback are all included in the profile of the tested samples.

### 3.3. Wheat Flour Gel Texture

Wheat flour gels prepared in the RVA were transferred to a 25 mL beaker and kept at room temperature overnight. In two penetration cycles, the gels were compressed at a speed of 0.5 mm/s to a distance of 10 mm, using a TA-TXT Texture Analyzer cylinder (Vienna Court, Lammas Road, UK). The following gel qualities were measured: hardness, cohesiveness, adhesiveness and gumminess, all of which were estimated according to Sandhu and Singh [34].

### 3.4. Dough Mixing Properties Using Micro-DoughLab

Using a 4.00 ± 0.01 g sample at 14% moisture basis after moisture correction, the Micro-DoughLab (Perten Instruments, Sidney, Australia) was used to establish the optimum water absorption capacity to obtain a peak of 500 FU. The samples were mixed at a speed of 63 rpm for 20 min at a temperature of 30 °C. At least three measurements were taken for each sample. Profiles were acquired for each mixture with and without CG or ZG (2% or 5%). The time it took for the dough to develop and how stable it was were both determined. The time difference between the point where the top of the curve first intersects the 500 BU line (arrival time) and the point where the top of the curve leaves the 500 BU line (departure line) is the dough stability. The mixing tolerance index (MTI) is the difference in BU between the top of the curve and five minutes afterwards. The degree of softening describes the difference between the consistency value of the curve center at the end of the developing time and the curve center 12 min after the developing time, where the farinograph quality number (FQN) is the length along the timeline from the beginning of water addition until the point where the center of the curve is 30 FU lower than at the development time.

### 3.5. Dough Extensibility

The method of Al-Saleh and Brennan [35] was used to determine dough extensibility. The dough was prepared in Micro-DoughLab according to the development time and correct water absorption after the flour or blend was replaced with 2% sodium chloride. The samples were prepared according to the instructions included with the Kieffer extensibility rig. The dough balls were then placed on a dough clamp and allowed to rest for 40 min at room temperature. The dough strips were placed on the sample plate, which was then loaded into the instrument’s sample holder. The Texture Analyzer (TA-XT plus, Stable Micro Systems, Godalming, Surrey, UK) was calibrated for a load cell of 50 kg to measure dough extensibility. The tensile test on the Kieffer rig was used to determine extensibility by the following settings: pre-test speed, 2.0 mm/s; test speed, 3.3 mm/s; post-test speed, 10.0 mm/s; distance75 mm; trigger force, auto-5 g; data rate acquisition, 200 point per second.

### 3.6. Solvent Retention Capacity (SRC)

The AACC method no. 56-11 was used to measure the solvent retention capacity (SRC) of the flour mixes [36]. Double distilled water, sugar (50% *v*/*v*), sodium bicarbonate (5% *v*/*v*), and lactic acid (5% *v*/*v*) were utilized as solvents. In 30 mL tubes, 25 mL of prepared solvents were added to 1.0 g of flour and centrifuged for 15 min at 1000× *g* (Fisherbrand^TM^ Refrigerated Centrifuge GT2, Hamburg, Germany). The weight of the precipitated gels was recorded after decanting the liquid, and the %SRC values for each solvent were calculated as follows:

(1) $$\mathrm{SRC}\;\left(\%\right)=\frac{wet\;pellet\left(g\right)}{\left[flour\;weight\left(g\right)-1\right]}\times \frac{86}{\left[100-flour\;moisture\;\left(\%\right)\right]}\times 100$$

### 3.7. Cookie Baking Procedure

Cookie baking was carried out according to AACC [36], method no. 10–50. The ingredients used were flour (control or with gum powders) at 14% moisture 225 g, sugar 64 g, shortening 64 g, 2.1 g sodium bicarbonate, 9.0 g dried egg whites, 8.9 g dextrose solution baking powder and 33 g water. After mixing all the ingredients according to the prescribed method, baking was performed at 205 °C for 10 min. The baked cookies were cooled and stored until further testing.

### 3.8. Physical Evaluation of Cookies

The AACC [36] method number 10–50 was used to determine the width, thickness, and spread factor of the cookies. The average width in mm was calculated after six cookies were put edge to edge. The average thickness in mm was calculated by stacking the same cookies on top of each other. The spread factor was estimated by dividing the width of the cookies by their thickness.

### 3.9. Texture Analysis of Cookies

The TA-TXT Texture Analyzer was used to determine the texture of the cookies (TA-XT plus, Stable Micro Systems, Godalming, Surrey, UK). The hardness and ability of the cookies to bend or snap (fracture ability) was tested using a three-point bending apparatus, as described by Abdel-Samie, et al. [37].

### 3.10. Cookie Color

The color values, such as L* (lightness), a* (redness) and b* (yellowness), of the cookie samples were determined using a Minolta color grader with a D65 light source [38].

### 3.11. Sensory Evaluation of Cookies

Staff and postgraduate students from King Saud University’s Department of Food Science and Nutrition were selected as panelists. The cookie samples were coded and randomly distributed to evaluators and evaluated on a 9-point hedonic scale, with 9.0 representing extremely good and 1.0 representing extremely poor, for qualities such as aroma, taste, texture, and general acceptability [39]. A sensory evaluation of the cookie samples was performed by a trained panel of judges. The panelists (12 members) were trained how to differentiate between the control cookie samples made with different formulations. The sensory panel consisted of panelists who were able to distinguish between the cookie samples that were similar and those that were different. All of the panelists were familiar with cookies because cookies are widely consumed in this society. The seven members of the panel were asked to compare and contrast the aroma, taste, texture, and overall acceptability of the samples and the control. Each of these sensory criteria was explained to the panelists, and they were given multiple opportunities to practice them.

### 3.12. Statistical Analysis

The data were analyzed using ANOVA, after the measurements were obtained in triplicate. The effects of CG and ZG on flour and cookie properties were investigated using a factorial design. The PASW^®^ Statistics 18 software was used and Duncan’s multiple range test was used to compare the means at *p* < 0.05.

## 4. Conclusions

The type and amount of gum in the mixture affected its water absorption. The blends with 5% of both gums had decreased water absorption, but the same blends had a longer dough development time, lower dough stability, and a substantially higher mixing tolerance index. The addition of gums, on the other hand, resulted in a much-increased water retention capacity. When compared to the control and the mix with CG, the inclusion of ZG improved dough extensibility. The gums enhanced the thickness and diameter of the cookies, but they also reduced the spread ratio and made the cookies less firm. Because of the gums, cookie fracturability was substantially higher. The cookies had a lower overall sensory acceptance than the control, but the gum-containing cookies had a considerably superior texture and up to 5% soluble fiber, due to the added gums. Considering the qualities of the gums used here, the current study leads to a better use of these wild fruits.

## Figures and Tables

**Table 1:** Dough development characteristics of flour gum blends.

	WA (%)	DDT (min)	Stability (min)	Softening (FU)	MTI (FU)	Quality Number FQN
Control (100% WF)	60.70 ± 0.14 ^a^	1.60 ± 0.08 ^c^	5.70 ± 0.22 ^a^	91.67 ± 2.36 ^d^	35.67 ± 4.19 ^e^	61.23 ± 0.95 ^a^
2% Cordia gum	59.57 ± 0.33 ^b^	1.13 ± 0.21 ^d^	2.20 ± 0.22 ^d^	100 ± 4.08 ^c^	70.00 ± 2.03 ^d^	52.30 ± 0.78 ^b^
5% Cordia gum	57.50 ± 0.24 ^c^	1.33 ± 0.05 ^d^	1.63 ± 0.05 ^e^	141.60 ± 2.33 ^a^	114.33 ± 3.04 ^a^	39.97 ± 0.05 ^e^
2% Ziziphus gum	60.87 ± 0.09 ^a^	3.43 ± 0.05 ^b^	3.50 ± 0.08 ^b^	126.30 ± 2.58 ^b^	89.00 ± 1.41 ^c^	46.20 ± 0.57 ^c^
5% Ziziphus gum	57.87 ± 0.09 ^c^	3.83 ± 0.21 ^a^	2.80 ± 0.01 ^c^	136.60 ± 6.23 ^a^	100.67 ± 0.94 ^b^	42.63 ± 0.39 ^d^

WF = wheat flour; WA = water absorption; DDT = dough development time; MTI = mixing tolerance index; FU = Farino units (DoughLab units); values followed by different letters in columns are significantly different at *p* ˂ 0.05.

**Table 2:** Solvent retention capacity properties of flour gum blends.

	WRC	SuSRC	SCRC	LARC
Control	74.50 ± 1.50 ^d^	130.50 ± 5.5 ^e^	101.50 ± 4.12 ^d^	143.50 ± 1.54 ^b^
2% Cordia	86.50 ± 0.50 ^c^	165.50 ± 6.50 ^c^	124.30 ± 4.25 ^b^	120.25 ± 2.13 ^c^
5% Cordia	137.12 ± 2.15 ^a^	295.50 ± 3.50 ^a^	137.21 ± 2.15 ^a^	97.50 ± 0.75 ^e^
2% Ziziphus	82.43 ± 1.02 ^c^	149.50 ± 3.50 ^d^	114.50 ± 1.50 ^c^	104.5 ± 8.50 ^d^
5% Ziziphus	104.50 ± 8.50 ^b^	212.50 ± 6.42 ^b^	132.10 ± 5.14 ^a^	174.12 ± 2.50 ^a^

WRC = Water retention capacity; SuSRC = Sucrose retention capacity; SCRC = Sodium carbonate retention capacity; LARC = lactic acid retention capacity. Values followed by different letters in columns are significantly different at *p* ˂ 0.05.

**Table 3:** Thickness, diameter and spread ration of the cookies.

	Thickness (mm)	Diameter (mm)	Spread Ratio
Control	8.59 ± 0.03 ^e^	53.50 ± 0.54 ^d^	6.23 ± 0.08 ^a^
2% Cordia	9.67 ± 0.03 ^c^	54.06 ± 0.55 ^c^	5.59 ± 0.07 ^c^
5% Cordia	10.26 ± 0.10 ^a^	56.44 ± 0.21 ^a^	5.50 ± 0.07 ^c^
2% Ziziphus	9.26 ± 0.08 ^d^	54.50 ± 0.27 ^c^	5.89 ± 0.08 ^b^
5% Ziziphus	9.98 ± 0.01 ^b^	55.39 ± 0.34 ^b^	5.55 ± 0.04 ^c^

Diameter/thickness = spread ratio. Values followed by different letters in columns are significantly different at *p* ˂ 0.05.

**Table 4:** Color, hardness and fracturability parameters of the cookies.

	Hardness (Grams)	Fracturability (mm)	L*	a*	b*
Control	2484.27 ± 25.34 ^a^	3.93 ± 0.04 ^e^	79.23 ± 0.02 ^a^	−5.49 ± 0.01 ^f^	30.05 ± 0.23 ^e^
2% Cordia	2238.22 ± 40.74 ^b^	5.27 ± 0.13 ^c^	69.62 ± 0.27 ^b^	1.25 ± 0.10 ^e^	34.47 ± 0.08 ^a^
5% Cordia	2016.83 ± 65.66 ^c^	5.51 ± 0.06 ^b^	65.00 ± 0.11 ^d^	3.65 ± 0.04 ^b^	31.47 ± 0.08 ^c^
2% Ziziphus	2022.09 ± 34.27 ^c^	5.90 ± 0.04 ^a^	64.23 ± 0.02 ^e^	4.04 ± 0.06 ^a^	33.49 ± 0.03 ^b^
5% Ziziphus	1908.67 ± 51.49 ^e^	5.08 ± 0.10 ^d^	67.93 ± 0.11 ^c^	2.52 ± 0.01 ^c^	33.55 ± 0.03 ^b^

L* = lightness; a* = green/red; b* = blue/yellow; values followed by different letters in columns are significantly different at *p* ˂ 0.05. Values followed by different letters in columns are significantly different at *p* ˂ 0.05.

**Table 5:** Sensory evaluation of cookies.

	Aroma	Taste	Texture	Color	Overall Acceptability
Control (100% WF) ^1^	8.10 ± 0.05 ^a^	7.89 ± 0.14 ^a^	8.01 ± 0.12 ^bc^	8.21 ± 0.12 ^a^	8.15 ± 0.15 ^a^
2% Cordia gum	7.05 ± 0.22 ^cd^	7.50 ± 0.21 ^c^	8.05 ± 0.22 ^bc^	7.85 ± 0.20 ^b^	7.75 ± 0.13 ^b^
5% Cordia gum	6.30 ± 0.14 ^d^	6.12 ± 0.15 ^e^	7.85 ± 0.25 ^c^	7.30 ± 0.15 ^c^	7.33 ± 0.09 ^cd^
2% Ziziphus gum	7.65 ± 0.16 ^b^	7.65 ± 0.21 ^bc^	8.30 ± 0.08 ^a^	7.90 ± 0.08 ^b^	7.56 ± 0.11 ^bc^
5% Ziziphus gum	6.52 ± 0.13 ^d^	6.83 ± 0.11 ^d^	8.41 ± 0.089 ^a^	8.30 ± 0.06 ^a^	7.42 ± 0.21 ^c^

^1^ Wheat flour; values followed by different letters in columns are significantly different at *p* ˂ 0.05. Values followed by different letters in columns are significantly different at *p* ˂ 0.05.

## Data Availability

Not applicable.
